# TWEAK-Fn14 Cytokine-Receptor Axis: A New Player of Myocardial Remodeling and Cardiac Failure

**DOI:** 10.3389/fimmu.2014.00050

**Published:** 2014-02-11

**Authors:** Tatyana Novoyatleva, Amna Sajjad, Felix B. Engel

**Affiliations:** ^1^Department of Cardiac Development and Remodelling, Max-Planck-Institute for Heart and Lung Research, Bad Nauheim, Germany; ^2^Government College University Faisalabad, Faisalabad, Pakistan; ^3^Department of Nephropathology, Experimental Renal and Cardiovascular Research, Institute of Pathology, University of Erlangen-Nürnberg, Erlangen, Germany

**Keywords:** cardiovascular disease, fibrosis, proliferation, hypertrophy, extracellular matrix, Toll-like receptors

## Abstract

Tumor necrosis factor (TNF) has been firmly established as a pathogenic factor in heart failure, a significant socio-economic burden. In this review, we will explore the role of other members of the TNF/TNF receptor superfamily (TNFSF/TNFRSF) in cardiovascular diseases (CVDs) focusing on TWEAK and its receptor Fn14, new players in myocardial remodeling and heart failure. The TWEAK/Fn14 pathway controls a variety of cellular activities such as proliferation, differentiation, and apoptosis and has diverse biological functions in pathological mechanisms like inflammation and fibrosis that are associated with CVDs. Furthermore, it has recently been shown that the TWEAK/Fn14 axis is a positive regulator of cardiac hypertrophy and that deletion of Fn14 receptor protects from right heart fibrosis and dysfunction. We discuss the potential use of the TWEAK/Fn14 axis as biomarker for CVDs as well as therapeutic target for future treatment of human heart failure based on supporting data from animal models and *in vitro* studies. Collectively, existing data strongly suggest the TWEAK/Fn14 axis as a potential new therapeutic target for achieving cardiac protection in patients with CVDs.

## Introduction

Most cardiovascular diseases (CVDs) result in heart failure due to the death of heart muscle cells, the cardiomyocytes. This leads to pathological remodeling, which triggers additional cardiomyocyte loss resulting finally in a diminished quality of life and inevitable in heart failure (1, 2). Thus, the most efficient way to prevent most CVDs appears to be preventing cardiomyocyte loss. One aim in cardiovascular medicine is therefore monitoring and targeting risk factors. These efforts resulted, for example, in the addition of antiplatelet therapy (3–6), introduction of reperfusion therapy with thrombolysis (7, 8), and acute percutaneous coronary intervention (9). The current optimal treatment regimen after the discovery that neurohormones contribute to the progression of heart failure is the use of angiotensin-converting-enzyme-inhibitors or angiotensin receptor blockers, beta-blockers, aldosterone antagonists as well as implantable automatic cardiac defibrillators. However, despite these advances, the prevalence of heart failure has increased in the last decades, remaining one of the leading causes of death worldwide (10, 11). This suggests that this treatment regimen does not target all pathological mechanisms in heart failure.

Already two decades ago, the observation of increased tumor necrosis factor (TNF) levels in patients with heart failure linked inflammation to CVDs (12). Meanwhile, a large number of reports have established the essential role of inflammatory cytokines in the progression of heart failure contributing to the processes of cardiac hypertrophy, fibrosis, and apoptosis (13–15). Recently, other TNFSF/TNFRSF members than TNF have been implicated in the pathophysiology of heart failure. Here, we review the role of the members of the TNFSF/TNFRSF in heart failure focusing on TWEAK and its receptor. In addition, we will explore their potential as biomarker for CVDs as well as therapeutic target for the future treatment of human heart failure.

## TWEAK and Its Cognate Receptor Fn14

The member TWEAK of the TNFSF was discovered in 1997 (16). Like the other ligands of the TNFSF, TWEAK is primarily synthesized as a type II transmembrane receptor and then further processed by a furin endoprotease into the soluble cytokine sTWEAK (16–18). Cells can co-express both plasma membrane-anchored and soluble TWEAK (19, 20). However, membrane-anchored TWEAK is due to its efficient cleavage, which is rarely detectable (e.g., in monocytes and macrophages) (21). TWEAK expression was reported in a wide variety of different tissues and cells, including tumor cell lines and specimens (16, 19, 22–30).

TWEAK is known to be the sole TNFSF that signals through the cell surface receptor Fn14, an unusual small TNFRSF member (31). Fn14 was discovered in 1999 as Fibroblast Growth Factor 1 (FGF1)-inducible, immediate-early response gene in murine NIH3T3 fibroblasts (32) and is induced by a large variety of other growth factors including FGF2, Platelet-Derived Growth Factor (PDGF), Epidermal Growth Factor (EGF) and Vascular Endothelial Growth Factor (VEGF) as well as cytokines such as tumor necrosis factor alpha (TNFα), Interleukin-1beta (IL-1β), Interferon gamma (IFNγ), and transforming growth factor-beta (TGF-β) (32–35). Fn14 is a type I transmembrane protein expressed on a broad variety of different cell types (18, 32, 33). It contains a single cysteine-rich domain (CRD) in its ectodomain while most other TNF receptors have two to six copies of this characteristic motif (36). The cytoplasmic domain of Fn14 contains only 28 amino acid residues lacking a death domain. Like other TNFRSF members lacking a death domain, Fn14 trimerizes upon ligand binding recruiting subsequently E3 ligase/adapter proteins of the tumor necrosis factor receptor-associated factor (TRAF) family to its cytoplasmic domain (21, 36). Several members of the TRAF family (TRAF1, TRAF2, TRAF3, and TRAF5) have been shown to be able to bind to Fn14 (37, 38).

## TWEAK/Fn14 Axis in Cardiomyocyte Proliferation

**Figure 1:**
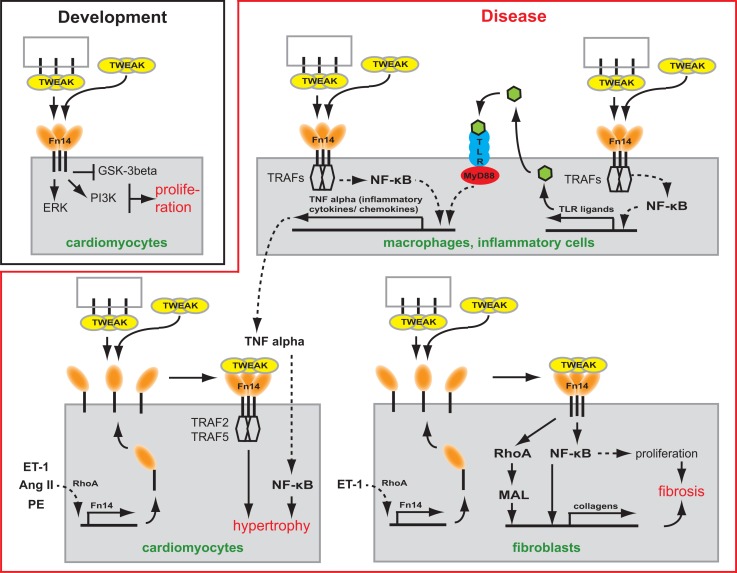
**Scheme depicting the potential role of TWEAK/Fn14 signaling in cardiac development and myocardial remodeling and cardiac failure**. TWEAK might be presented to Fn14 as membrane-bound or secreted form. TWEAK stimulation induces *in vitro* proliferation of neonatal cardiomyocytes. Thus, TWEAK/Fn14 signaling might contribute to developmental heart growth. In CVDs, it has been shown that TWEAK has the potential to affect inflammatory cells, cardiomyocytes as well as fibroblasts. In inflammatory cells, TWEAK can enhance secretion of inflammatory cytokines/chemokines by enhancing their expression directly or by increasing the expression of TLR ligands. In cardiomyocytes, TWEAK induces via TRAF hypertrophy. In fibroblast, TWEAK induces the expression of collagens via RhoA and NF-κB and stimulates via NF-κB proliferation leading to cardiac fibrosis.

The heart grows during embryonic development mainly due to cardiomyocyte proliferation. Shortly after birth, however, cardiomyocytes stop to proliferate and the heart continues to grow through the increase in cardiomyocyte cell size (i.e., hypertrophy) (39). Consistent with its role in proliferation in a number of cell types, such as smooth muscle cells (40), myoblasts (28, 41), astrocytes (42), liver progenitor cells (29), epithelial (43), and tubular cells (44), Fn14 expression correlates with the rate of cardiomyocyte proliferation during heart development (45). However, neither Fn14 nor TWEAK knockout mice exhibit a heart phenotype suggesting that the TWEAK/Fn14 axis is not essential for cardiomyocyte proliferation or heart development (28–30). Yet, TWEAK stimulation of neonatal rat cardiomyocytes, expressing Fn14 endogenously, induced cardiomyocyte proliferation (45). TWEAK activated extracellular signal-regulated kinase (ERK) and phosphatidylinositol 3-kinase (PI3K) but not p38 mitogen-activated kinase (p38) signaling. In addition, TWEAK inhibited glycogen synthase kinase-3beta (GSK-3beta) (Figure 1) (45). The effect of TWEAK on several pathways has been described also for other cell types. In tubular cells, TWEAK activated, for example, ERK, p38, PI3K, and NF-κB signaling (44). TWEAK-induced proliferation in tubular cells and cardiomyocytes was prevented by inhibitors of ERK and PI3K (44, 45). In contrast, inhibition of p38 blocked only tubular cell proliferation. A general role of TWEAK-induced NF-κB signaling in cell proliferation remains unclear as it has not yet been determined in cardiomyocytes. In tubular cells, inhibition of NF-κB signaling blocked proliferation.

In contrast to neonatal rat cardiomyocytes, TWEAK had a negligible effect on adult cardiomyocyte proliferation, possibly due to the developmental downregulation of Fn14. However, ectopic expression of Fn14 enabled TWEAK-induced DNA synthesis in adult cardiomyocytes. To date, activation of TWEAK/Fn14 signaling is by far the most potent inducer of adult cardiomyocyte cell cycle re-entry but fails to promote progression into mitosis (45). This is important, as induction of cardiomyocyte proliferation is considered to be a potential future therapy to CVDs. Adult zebrafish and newt as well as newborn mice can all regenerate their heart through cardiomyocyte proliferation (46–48). Moreover, several studies have demonstrated that adult mammalian cardiomyocyte cell division can be induced, even though induction efficiency is relatively low (49, 50). Finally, recent reports utilizing carbon-14 isotope labeling due to atomic bomb tests in the 60s suggest that also human adult mammalian cardiomyocytes, at least a sub-set, might maintain the competence to proliferate (51). Thus, in the future it will be important to elucidate the TWEAK-mediated signaling that induces rat neonatal cardiomyocyte proliferation and to determine if reinstatement of these signaling modalities allows also adult mammalian cardiomyocyte proliferation.

## The TWEAK/Fn14 Signaling Promotes Cardiac Hypertrophy and Heart Failure

Pathological cardiac hypertrophy is a key risk factor for heart failure. Cardiac hypertrophy describes the enlargement of the heart due to the increase in cell size of cardiomyocytes. For example, physical exercise and pregnancy can lead to cardiac hypertrophy (52). This form of hypertrophy is considered physiological cardiac hypertrophy as heart function is not affected or improved. In contrast, hypertrophy induced by chronic pressure or volume overload results under certain disease conditions such as hypertension, valvular heart disease, and coronary artery disease, in cardiac dysfunction or heart failure (52). Thus, it is called pathological cardiac hypertrophy.

Tumor necrosis factor alpha was the first member of the TNFSF shown to induce cardiomyocyte hypertrophy (53). Cardiomyocyte-specific overexpression as well as infusion of TNFα causes dilated cardiomyopathy (DCM) suggesting that both circulating and locally produced TNFα induces myocardial dysfunction (54, 55). In recent years, animal experiments have suggested that also other TNFSF ligands can mediate cardiac hypertrophy and heart failure. For example, transgenic overexpression of FasL (TNFSF6) resulted in cardiac hypertrophy with pro-inflammatory consequences (56). That also the TWEAK/Fn14 axis is involved in cardiac hypertrophy was supported by the discovery that transgenic overexpression of full length-TWEAK (fl-TWEAK) in mice resulted in DCM with markedly increased heart to body weight ratio and severe cardiac dysfunction. Moreover, cardiomyocytes from fl-TWEAK-overexpressing mice displayed cellular hypertrophy characterized by pronounced cellular elongation (57). It has also been demonstrated that endogenous Fn14 is required for cardiac hypertrophy. Fn14 deletion attenuated right ventricular (RV) hypertrophy caused by pulmonary artery banding (PAB), a mouse model of pressure-overload-induced RV hypertrophy while TWEAK/Fn14 signaling promoted cardiomyocyte hypertrophy *in vitro* (58). However, the upstream and downstream signaling pathways regulating TWEAK/Fn14-mediated hypertrophy *in vivo* remain unclear. It has been shown that hypertrophic agonists including Angiotensin II (Ang II), Phenylephrine (PE), and Endothelin-1 (ET-1) induce Fn14 expression (59). Furthermore, TRAF2 and TRAF5, possible downstream targets of TWEAK/Fn14 signaling, have been implicated in cardiac hypertrophy (Figure 1). Cardiomyocyte-specific TRAF2 transgenic mice developed a time-dependent increase in cardiac hypertrophy, left ventricular (LV) dilation, and adverse LV remodeling, and a significant decrease in heart function (60). Moreover, deficiency of TRAF5 substantially aggravated cardiac hypertrophy and cardiac dysfunction in response to pressure overload after transverse aortic constriction (TAC) (61).

## TWEAK is an Extracellular Matrix Modulating Factor in the Heart

Heart failure describes a condition when the heart fails to pump sufficient blood to meet the metabolic demand of the body. It is caused by the loss of cardiomyocytes through necrosis, apoptosis, and autophagy (62, 63), which results in hypertrophy, myocardial fibrosis (fibrillar collagen deposition), and maladaptive extracellular matrix (ECM) remodeling, all characteristics of end-stage heart failure (64). ECM remodeling leads to cardiomyocyte slippage, ventricular dilatation, increased ventricular stiffness as well as impaired diastolic and systolic function (65).

As described above, TNFSF members are involved in the early stages of CVDs such as increased inflammation and hypertrophy (56, 66). However, there is clear evidence that TNFSF members such as TNFα or FasL are also directly involved in myocardial fibrosis (56, 66). First evidence that the TWEAK/Fn14 axis may play a role in ECM remodeling came from the analysis of fl-TWEAK overexpressing mice. These mice develop DCM exhibiting progressive myocardial and perivascular fibrosis (57). Recently, it has been shown that PAB-induced fibrosis was significantly reduced in Fn14 global knockout mice (30). Cell culture experiments demonstrated that TWEAK/Fn14 signaling promotes cardiac mouse fibroblast proliferation and collagen synthesis (30), major sources of fibrillar collagen in the heart under pathophysiological conditions (67, 68). Collagen expression induced by TWEAK/Fn14 signaling was mediated via RhoA-dependent nuclear translocation of the myocardin-related transcription factor-A (MRTF-A)/MAL. Interestingly, upregulation of Fn14 expression in cardiomyocytes due to stretch or stimulation with Ang II or norepinephrine was mediated by RhoA/ROCK signaling, too (69). Furthermore, Chen and colleagues independently demonstrated that TWEAK induces proliferation and collagen synthesis of rat cardiac fibroblasts (70). However, they showed that proliferation and enhancement of collagen synthesis was mediated by the activation of NF-κB signaling (Figure 1). Collectively, these data demonstrate that the TWEAK/Fn14 axis is involved in cardiac ECM remodeling. Importantly, Fn14 knockout mice were protected from PAB-induced RV dysfunction (30, 58) as well as TWEAK-induced cardiac dysfunction and dilation (57).

An essential prerequisite for the formation of fibrotic scar tissue and ECM remodeling is, besides the elevated production of ECM proteins, the expression of matrix metalloproteinases (MMPs) and tissue inhibitors of metalloproteinases (TIMPs) (71–73). Previously, it has been demonstrated that TNFα-induced cardiac remodeling and dysfunction depends on MMP activation (74). In addition, it has been shown that also FasL and other TNFSF members such as LIGHT, RANKL, and CD40L can potentially regulate MMP activity (75–77). Finally, both TNFα and FasL overexpression is associated with increased levels of TGF-β1, an important inducer of myocardial fibrosis (56, 66). Taken together, these data suggest that besides TNFα several other members of the TNFSF might play important roles in ECM remodeling in cardiac disease.

## TWEAK and Other TNFSF Signal via NF-κB

NF-κB has been shown to be cardio-protective (78). However, prolonged activation of NF-κB appears to promote heart failure. For example, cardiomyocyte-specific IκB kinase (IKK)/NF-κB activation induces reversible inflammatory cardiomyopathy and heart failure (79). In addition, it has been demonstrated in animal models, such as TAC-induced pressure-overload (80, 81) and monocrotaline (MCT)-induced RV hypertrophy (82), that inhibition of NF-kB signaling prevents heart failure (cardiac hypertrophy and/or cardiac remodeling).

NF-κB transcription factors are activated through cytokines, pathogens, injuries, and other stressful conditions. Mammalian cells express five NF-κB family members (RelA, RelB, c-Rel, NF-κB2/p100/p52, and NF-κB1/p105/p50) (83–85), that regulate the expression of a large variety of genes which are involved in a number of processes like inflammatory and immune responses of the cell, cell growth, and development. In unstimulated cells, NF-κB is bound to an inhibitory protein, IκB. Binding to IκB masks the nuclear localization signal of NF-κB, sequesters the NF-κB/IκB complex in the cytoplasm, and prevents NF-κB from binding to DNA. Canonical NF-κB signaling culminates in the activation of IKK, which phosphorylates the inhibitory IκB subunit of the NF-κB/IκB complex in the cytoplasm resulting in the proteasomal degradation of IκB. This releases NF-κB resulting in the translocation of NF-κB into the nucleus. In contrast, the non-canonical NF-κB signaling pathway mediates activation of the p52/RelB NF-κB complex. This NF-κB pathway relies on the inducible processing of NF-κB2 precursor protein, as opposed to the degradation of IκBα. A central signaling component of the non-canonical NF-κB pathway is NF-κB-inducing kinase (NIK), which functions together with the inhibitor of NF-κB kinase α (IKKα), to induce phosphorylation-dependent ubiquitination and processing of NF-κB2. Under normal conditions, NIK is continuously degraded. In response to signals mediated by a sub-set of TNFSF members such as Lymphotoxin-β (LT-β), B-cell activating factor (BAFF), and CD40 ligand (CD40L) (86–89), NIK becomes stabilized leading to the activation of non-canonical NF-κB (90, 91).

TWEAK/Fn14 axis has been shown to activate several different signaling cascades, though activation of NF-κB signaling appears to be the major and predominant cellular response through which TWEAK/Fn14 signals. TWEAK/Fn14 has been demonstrated to activate canonical NF-κB signaling in a large variety of cell types (38, 41, 70, 92–96). Interestingly, TWEAK can also signal via Fn14 through the non-canonical NF-κB pathway, which is dependent on the TRAF-binding site of Fn14 as well as TRAF2 and TRAF5 (97). Membrane-bound and oligomerized sTWEAK are superior to soluble TWEAK trimers in regard to the activation of the classical NF-κB pathway. In contrast, both TWEAK variants are equally potent inducers of the non-canonical NF-κB pathway (98). That TWEAK/Fn14 mediates its detrimental effect on heart function at least in part through NF-κB signaling has been supported by several studies. TWEAK-induced proliferation and collagen synthesis of rat cardiac fibroblasts *in vitro* was mediated by the activation of NF-κB signaling (70). Moreover, DCM induced through elevated circulating TWEAK levels occurred via an FN14-TRAF2-NF-κB-dependent signaling pathway (99). In addition, cardiomyocyte-specific TRAF2 overexpressing mice provoked adverse cardiac remodeling associated with elevated NF-κB signaling (60). These data suggest that the members of the TNFSF mediate their detrimental effects in the heart through TNFRSF members via TRAF2, which is associated directly or indirectly with the majority of TNFSFR members expressed in the heart (TNFR1, TNFR2, RANK, and Fn14), through non-canonical NF-κB signaling (77).

## Potential Interactions of Toll-Like Receptors and TWEAK/Fn14 Signaling in CVD

Toll-like receptors (TLRs) are a family of single, membrane-spanning, non-catalytic receptors, which are expressed on various immune cells, such as macrophages, dendritic cells, and neutrophils, as well as on non-immune cells, such as fibroblast cells and epithelial cells. Most commonly, they are known as key activators of the innate immune system as they are responsible for the synthesis and secretion of various inflammatory cytokines by the cells of this system (100). Upon detection of distinct pathogen-associated molecular patterns (PAMPs) of protozoa, virus, and bacteria origin, different members of the TLR family activate signaling pathways that result in the activation of NF-κB-dependent and interferon regulatory factor (IRF)-dependent molecular mechanisms. In addition, TLRs may also be activated by endogenous ligands named damage-associated molecular patterns (DAMPs), which allow the immune system to sense tissue injury in the absence of an infection.

TLR2 and TLR4 activation resulting in NF-κB-dependent release of inflammatory cytokines plays an important role in CVD (101–103). For example, it has been demonstrated that TLR2-deficient mice exhibit higher fractional shortening and survival after myocardial infarction in comparison to wild-type animals (104). In addition, both knockout of TLR2 and inhibition of TLR2 by neutralizing antibodies significantly reduced Ang II-induced cardiac fibrosis, which was associated with a reduction in the infiltration of macrophages, the production of inflammatory cytokines and chemokines, and the activation of NF-κB (103). However, TLR2 deletion in a hypertrophy model (TAC) revealed that TLR2 is required for adaptive cardiac hypertrophy through IL-1β upregulation via NF-κB activation (102). Similar to TLR2, TLR4-deficient mice show after myocardial infarction enhanced LV function and improved remodeling leading to significantly increased survival of TLR4-deficient mice (105, 106). Finally, adenoviral overexpression of dominant-negative MyD88, a common adaptor of TLR2 and TLR4, significantly reduced cardiac hypertrophy and cardiac fibrosis in an aortic constriction model improving cardiac function (107). Taken together there is accumulating evidence for detrimental effects of TLR signaling on cardiac remodeling, cardiac function, and fibrosis upon injury (Figure 1) (108).

Interestingly, it has recently been indicated that TWEAK has the ability to potentiate the pro-inflammatory effects of TLR ligands. For instance, TWEAK has been shown to cooperate with the TLR2 ligand Pam_3_CysSK_4_ on the stimulation of IL-8 synthesis by epithelial cells (109). Furthermore, TWEAK is able to stimulate the secretion of HMGB1 (110, 111), another postulated TLR ligand, that contributes to the inflammation in various injury models via signaling through TLR2, TLR4, and RAGE in inflammatory cells (112, 113). Collectively, these data suggest that the TWEAK/Fn14 signaling pathway may also interact with TLR signaling in promoting acute inflammation in CVD.

## Prognostic Value of TWEAK/Fn14 Expression for Heart Failure

During recent years, evidence has accumulated that other members of the TNFRSF/TNFSF than TNFα/TNFR might play important roles in the development and progression of heart failure as they are regulated in both experimental and clinical heart failure. Expression analyses of TNFSF ligands and co-stimulatory molecules have revealed that cardiomyocytes of patients with acute myocarditis and DCM express high levels of CD27L, CD30L, and 4-1BBL and exhibit weak to moderate expression of OX40L (114). In heart failure post-myocardial infarction, RANKL was upregulated in both fibroblasts and cardiomyocytes (77). In addition to cardiac cells, elevated expression of TNFSF members were also observed in T lymphocytes in DCM (CD40L) (115) and peripheral blood mononuclear cells in chronic heart failure (4-1BBL, APRIL, CD27L, CD40L, FasL, LIGHT, and TRAIL-receptor 4) (116). Notably, receptors for several of these ligands (e.g., FasL, LIGHT, TNFα, RANKL, and TRAIL) have been reported to be expressed in the heart and enhanced levels of some of these TNF-related molecules also have been found within the failing myocardium (e.g., RANKL, OPG) (77, 117–124). That the members of the TNFSF, can be utilized as prognostic markers, is exemplified by OPG (125), whose plasma level correlated in apparently healthy patients with greater LV mass and lower LV ejection fraction (126). OPG also has been shown to be a reliable predictor of long-term mortality and heart failure development in patients with acute coronary syndrome (127), all-cause mortality in patients with symptomatic severe aortic stenosis (128) or even mixed etiology (129), and with hospitalization of patients with ischemic heart failure due to worsening of heart failure. Another example is BAFF, which is elevated in patients with acute myocardial infarction predicting increased risk of death or recurrent myocardial infarction (130).

In contrast to the poor prognosis found in relation to elevated TNFα levels in heart failure, increased levels of sTWEAK appear to be a good predictor of an adverse short-term outcome after severe type of myocardial infarction (ST-elevation myocardial infarction, STEMI) correlating with hospital duration time of the patients (131). In contrast, TWEAK protein levels were lowered in patients with chronic stable heart failure (132) or advanced non-ischemic heart failure (133). sTWEAK levels were inversely correlated with the severity of the disease and allowed prediction of patient’s mortality, respectively. Importantly, the predictive value was also verified after adjustment for clinical and biochemical variables including the state of the art biomarker, NT-proBNP.

However, sTWEAK alone appears not to be an optimal predictor of heart disease in general as Fn14 gene expression, in contrast to other members of the TNFRSF, is highly regulated *in vivo*. Under physiological conditions, Fn14 is expressed at relatively low levels but its expression is elevated in several experimental models of injury and inflammation (18, 28, 29, 94, 134, 135). The predictive value of sTWEAK levels is also complicated by the fact that TWEAK and Fn14 can be expressed by a wide variety of cell types. Both are expressed in cardiomyocytes (30, 45, 57, 131) and cardiac fibroblasts (30, 70). In addition, their expression was observed also for macrophages and smooth muscle cells of carotid atherosclerotic plaques (134). Finally, TWEAK is also expressed in endothelial cells of coronary arteries (59) and Fn14 expression was upregulated in proliferating endothelial and smooth muscle cells of injured rat arteries (18). Thus, changed levels of plasma sTWEAK might be difficult to interpret. For example, plasma sTWEAK levels are decreased in patients with pulmonary arterial hypertension (PAH), which results in RV failure (136). This might suggest that sTWEAK has positive adaptive functions. However, animal experiments have demonstrated that Fn14 expression in the heart is highly upregulated after PAB- or MCT-induced PAH (30). In these animal models, TWEAK blood plasma levels were unchanged (PAB) or significantly reduced (MCT) while TWEAK mRNA expression in RVs was elevated. Thus, reduced TWEAK blood levels might be due sequestration of circulating TWEAK by the upregulated Fn14 receptor or might be a compensatory mechanism to protect from the consequences of Fn14 activation. Collectively, sTWEAK appears to be a promising biomarker if combined with clinical parameters.

## Blocking of Fn14 Signaling as Potential Therapeutic Approach

In addition to novel candidates for new biomarkers, several TNF-related molecules also could be attractive targets for cardiac therapy. Cell culture as well as *in vivo* experiments have indicated that TWEAK/Fn14 signaling is involved in cardiac hypertrophy, cardiac remodeling, and heart failure, identifying TWEAK and Fn14 as promising targets to treat CVDs (30, 45, 58, 59, 69, 70, 99, 137). Targeting TWEAK and Fn14 has been also considered in various other pathophysiological conditions. Blocking of TWEAK or Fn14 has successfully been demonstrated to be beneficial in preclinical models of Collagen-Induced Arthritis (138, 139), Experimental Autoimmune Encephalitis (140), Middle Cerebral Artery Occlusion (94, 135, 141), Ischemia Reperfusion Injury (142), and atherosclerosis (143, 144). Furthermore, therapeutical efficacies of TWEAK and Fn14 blocking antibodies were determined in tumor growth inhibition assays, utilizing TWEAK and Fn14-expressing human esophageal and pancreatic cell lines, as well as in a murine gastrointestestinal cancer model (145). Anti-TWEAK and anti-Fn14-specific antibodies are at the moment under clinical investigations in phase I studies in patients suffering on Rheumatoid Arthritis, lupus or solid tumors (http://clinicaltrials.gov/; NCT00771329, NCT001499355 and NCT00738764). Additionally to the usage of antibodies, the employment of the fusion proteins Fn14-Fc and Fc-TWEAK as well as soluble TWEAK provide alternative approaches (137). Taken together, therapies targeting TWEAK and/or Fn14 appear to be a realistic approach and thus warrant future preclinical studies.

## Concluding Remarks

The members of the TNFSF and TNFRSF have been shown to be involved in the progression of CVDs to heart failure and thus they appear to be promising prognostic and therapeutic targets. However, the past has shown that correlations of cytokine blood levels to heart disease can be misleading (146). For example, TNFα mediates both adaptive and maladaptive effects on the myocardium. On the one hand it activates via NF-κB expression of anti-apoptotic and cytoprotective genes, but on the other hand it induces also inflammation (147). This explains why clinical trials with anti-TNF therapies were disappointing although overexpression of TNF, which is positively correlated with heart failure in patients, leads to experimental heart failure (146, 148). Thus, it will be important to consider this issue when designing new treatment strategies in heart failure that target members of the TNFSF or TNFRSF.

Based on the disappointing results from anti-TNF trials, the TWEAK/Fn14 axis may represent new targets for heart failure therapies. Fn14 appears to be an excellent therapeutic target as Fn14 knockout mice are viable and show no obvious phenotype under physiological conditions. In addition, Fn14 is upregulated in the myocardium of diseased hearts (30). However, at present the precise role of the TWEAK/Fn14 axis is still poorly understood, and it is unclear whether it has a positive, adaptive role in cardiac disease. One important issue is that the TWEAK/Fn14 axis regulates the behavior of several different cell types. Yet, genetic models inhibiting the TWEAK/Fn14 axis were beneficial in experimental models of heart disease (30, 58). Thus, it is important to test next treatment strategies such as anti-TWEAK antibodies in experimental heart failure models.

Cardiovascular diseases resulting in heart failure are highly complex diseases. Thus, in an ideal case a biomarker should be involved in several pathways of these multiple-pathway diseases reflecting several important pathophysiologies such as hypertrophy, fibrosis, remodeling, and inflammation. As the members of the TNFSF are involved in several of these processes, they appear to be promising biomarkers. However, it might be impudent to assume that a single member of the TNFSF is sufficient. For example, although NT-proBNP is a strong biomarker in heart failure it has recently been shown in patients with symptomatic aortic stenosis that the combination of high levels of both OPG and NT-proBNP was strongly associated with all-cause mortality, thus providing more information together than when either of these markers was used alone (128). Yet, a combination of TNFSF members that provides a “signature of disease” appears likely to be a suitable tool for risk prediction.

## Author Contributions

Tatyana Novoyatleva and Felix B. Engel wrote the manuscript. Felix B. Engel generated Figure 1. Amna Sajjad contributed to the literature search. All authors proof read the manuscript.

## Conflict of Interest Statement

The authors declare that the research was conducted in the absence of any commercial or financial relationships that could be construed as a potential conflict of interest.
